# Timing and Modality of Hemorrhoidal Prolapse Impact on Patients’ Quality of Life

**DOI:** 10.3390/jcm13133946

**Published:** 2024-07-05

**Authors:** Carlo Ratto, Angelo Parello, Angelo Alessandro Marra, Paola Campennì, Veronica De Simone, Francesco Litta

**Affiliations:** 1 Proctology and Pelvic Floor Surgery Unit, Ospedale Isola Tiberina—Gemelli Isola, 00186 Rome, Italy; angelo.parello@fbf-isola.it (A.P.); angeloalessandromarr@libero.it (A.A.M.); paola.campenni@fbf-isola.it (P.C.); veronica.desimone@fbf-isola.it (V.D.S.); francesco.litta@fbf-isola.it (F.L.); 2 Department of Medicine and Translational Surgery, Catholic University of the Sacred Hearth, 00168 Rome, Italy

**Keywords:** hemorrhoidal prolapse, quality of life, symptoms

## Abstract

**Background**: The aim of this study was to assess whether the frequency and presentation modality of hemorrhoidal prolapse may have an impact on patients’ quality of life, leading to a different categorization of patients. **Methods**: A consecutive series of patients affected by primary hemorrhoidal disease were administered specific questionnaires to assess the severity of symptoms and their quality of life. The frequency/modality of prolapse was also assessed, and the classification of the patients into five categories was hypothesized. The severity of disease was assessed using a validated patient-reported score, while the health-related quality of life was evaluated with the Short Health Scale for hemorrhoidal disease. **Results**: A total of 122 patients were enrolled. The evaluation of the prolapse modality led to the following classification: type I, 5 patients (4.1%); type II, 9 (7.4%), type IIIa, 48 (39.3%); type IIIb, 52 (42.6%); and type IV, 8 (6.6%). The mean total hemorrhoidal disease score was 9.8 ± 3.3, while the mean total Short Health Scale score was 18.6 ± 5.2. Both scores progressively increased in the five types of prolapse identified, thus showing a worsening of symptoms and quality of life related to the increase in the frequency and modality of prolapse. The assessment of the quality of life showed that all four domains of the Short Health Scale score and the total score were significantly worse in group IIIb compared to IIIa. **Conclusions**: The frequency and modality of hemorrhoidal prolapse has an impact on the quality of life and allows the identification of new types of patients.

## 1. Introduction

Hemorrhoidal disease (HD) is one of the most common proctological conditions [1], with a great impact on patients’ quality of life [2]. The first-line treatment of HD is usually conservative, while surgery is required when it fails or under special conditions [3,4]. In both clinical practice and scientific reports, the management of HD is usually based on the degree of the disease. The traditional Goligher classification system [5] has been widely accepted and adopted in the vast scientific literature on HD and almost all international guidelines for HD treatment [3,4]. This system considers the anatomical alterations in the hemorrhoidal piles and the two main consequent HD-related symptoms (i.e., bleeding and prolapse). In the Goligher classification, special regard is dedicated to the hemorrhoidal prolapse’s spontaneous, manual, or impossible reduction, which configures grades II, III, and IV, respectively. So far, conservative options or in-office procedures have been most frequently indicated in Goligher I- or II-degree disease, while surgery has been mostly recommended in III- and IV-degree HD, when a more severe prolapse is the predominant symptom [6]. However, the Goligher criteria have shown several limitations, mainly their poor correlation with the severity and frequency of HD symptoms [7] and a lack of consideration of other disease symptoms [8]. Consequently, a lot of controversy has arisen in daily practice as well as in guidelines, especially following the recent introduction of new treatment procedures. For these reasons, several classifications have been proposed over the last few years, but their complexity and scarce adherence in real clinical use have made them unsatisfactory for routine practice [9,10,11]. More recently, the importance of assessing the severity of HD and its impact on a patient’s quality of life (QoL) has emerged [12]; moreover, an established diagnostic tool called PROMs (patient-reported outcome measures) has recently been attempted in HD [13].

In consideration of the role played by hemorrhoidal prolapses in HD evolution, diagnostic, classification, and patient management, in this study, special regard was reserved to the assessment of the frequency and conditions of hemorrhoidal prolapse occurrence, related to the patients’ QoL, to discover whether these measures could differentiate patients with more reliable criteria.

## 2. Materials and Methods

Study Setting and Approval

This was a prospective, monocentric observational study conducted in the Proctology Unit of the Fondazione Policlinico Universitario A. Gemelli IRCCS in Rome, Italy, between May 2020 and April 2021. The local Ethics Committee approved this study (Prot. ID 3339). All patients signed a written informed consent form.

Inclusion and Exclusion Criteria

A consecutive series of patients referred to our unit and affected by HD were evaluated by an assessment of their medical history (specifically including their HD characterization) and physical examination (including digital anorectal examination and anoscopy). Moreover, all patients were prescribed an endoscopic colorectal evaluation according to the common guidelines [3].

The criteria for inclusion in this study were an age between 18 and 80 years, primary HD, consent to participate in this study, and attending all scheduled follow-up visits. The exclusion criteria were the following: recurrent HD, previous ambulatory or surgical treatment of HD, colorectal or anal cancer, obstructive defecation syndrome, irritable bowel syndrome, chronic inflammatory bowel disease, coagulation disorders, other proctologic diseases (including anal abscess/fistula, anal fissure, or acute hemorrhoidal thrombosis), and pregnancy. 

This study did not alter the scheduled treatment for the patients, who were therefore treated according to the usual modalities of our unit.

Data Collection and Measurements
Hemorrhoidal Prolapse Modality

To assess the hemorrhoidal prolapse, the patients were asked a question, investigating if the prolapse was reducible either spontaneously or manually or irreducible and when the prolapse usually occurred (either only at defecation or also not at defecation) (Table 1).

Based on the seven potential answers, the following classification of patients into 5 types was hypothesized:

- type I, if a patient had no prolapse at all (answer #1);
- type II, if the prolapse occurred only at defecation, with prompt spontaneous reduction (answer #2);
- type III-a, if the prolapse occurred only at defecation but needing time for spontaneous reduction (answer #3) or needing manual reduction (answer #4);
- type III-b, if the prolapse occurred not only at defecation but also on other occasions during the day, reducing either spontaneously (answer #5) or by manual maneuver (answer #6);
- type IV, if the prolapse was not reducible because fixed outside the anus (answer #7).

Symptoms of HD

Before the physical examination (including digital anorectal examination and anoscopy), all the patients enrolled were requested to fill the questionnaire for the Hemorrhoidal Disease Symptom Score (HDSS), as proposed by Rorvik et al. [12], a patient-reported score based on five cardinal symptoms, including pain, itching, bleeding, soiling, and prolapse (Table 2). Each symptom was assessed specifically concerning its frequency, as follows: 0 = never; 1 = less than once a month; 2 = less than once a week; 3 = 1–6 days per week; and 4 = every day or always. The total score, therefore, ranged from 0 (no symptoms at all) to 20 (daily occurrence of all symptoms).

Quality of life

The health-related QoL was evaluated with the Short Health Scale for HD (SHS_HD_), as described by Rorvik et al. [12] (Table 2). This tool examines the impact of HD on patients’ QoL through 4 questions focusing on symptom severity, interference with daily activities, concern caused by the symptoms, and general well-being. Each item is graded using a 7-point Likert scale (from 1 = no symptoms to 7 = most severe symptoms), with a total score ranging from 4 to 28.

Statistical analysis

Sample size calculation was not performed, in line with the COSMIN guidelines, which consider the sample sizes for the assessment of the measurement properties as excellent if the number of patients enrolled is >100, good if >50, and poor if >40 [12,14].

Continuous data were analyzed as the mean (with standard deviations, SD) and compared using the Mann–Whitney test and the Kruskal–Wallis test. Categorical data were analyzed as frequencies and percentages and compared using the chi-square test. A *p* < 0.05 was considered statistically significant. All data recorded were collected with an Excel spreadsheet and analyzed with the SPSS statistical version 21.0 for Windows software (SPSS, Chicago, IL, USA).

## 3. Results

A total of 122 patients (69 males, mean age 50.0 ± 12.7 years) affected by primary HD were consecutively enrolled in this study. After the clinical and physical examinations, the patients were classified according to the Goligher criteria as follows: I-degree, 16 cases (13.1%); II-degree, 42 cases (34.4%); III-degree, 54 cases (44.3%); and IV-degree, 10 cases (8.2%).

### 3.1. Hemorrhoidal Prolapse Modality

Prolapse occurred as follows: never in 4.1% of cases; only at defecation, with prompt spontaneous reduction after defecation in 7.4% of cases; only at defecation but the patients either had to wait a long time to obtain a spontaneous reduction (18.9%) or it had to be reduced manually (20.5%); not only at defecation but also far from it, needing a long time for a spontaneous reduction in 18.0% of cases or having to opt for a manual reduction in 24.6% of cases; and fixed and not reducible in 6.6% of cases (Table 3). Based on these responses, the patients were classified as follows: type I, 5 (4.1%) patients; type II, 9 (7.4%); type IIIa, 48 (39.3%); type IIIb, 52 (42.6%); and type IV, 8 (6.6%).

### 3.2. HDSS and SHS_HD_

The symptoms reported by the patients and their QoL assessment are reported in Table 3: the mean total HDSS was 9.8 ± 3.3, while the mean total SHS_HD_ was 18.6 ± 5.2.

**Figure 1:**
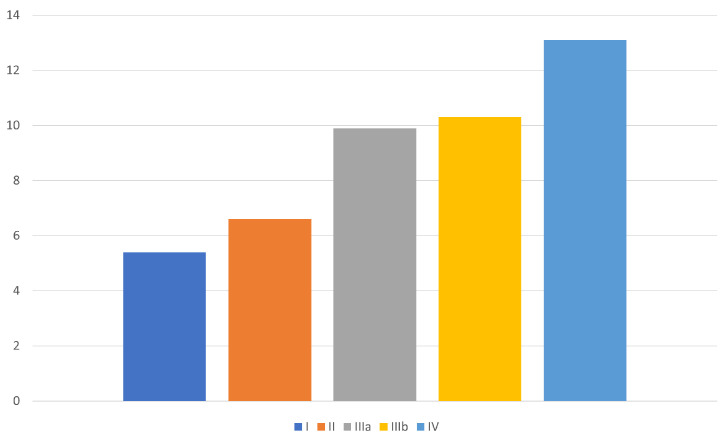
The Hemorrhoidal Disease Symptom Score (HDSS)^11^ progressively increased in the five types of prolapse identified, thus showing a worsening of symptoms related to the increase in the frequency of prolapse and the modality of prolapse reduction.

The HDSS progressively increased in the five types of prolapse identified, thus showing a worsening of symptoms related to the increase in the frequency of prolapse and the modality of prolapse reduction (Figure 1). 

**Figure 2:**
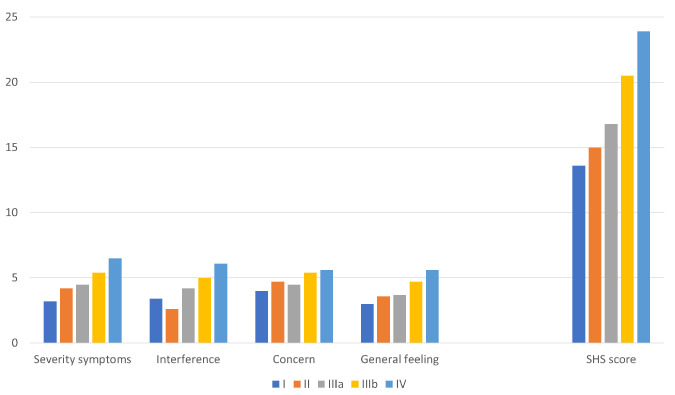
Health-related quality of life evaluated with the Short Health Scale for HD (SHS_HD_)^11^: the total score and its four domains, progressively increasing in relation to the five types of prolapse identified.

Similarly, the total SHS_HD_ and its four domains progressively increased in relation to the five types of prolapse identified (Figure 2).

### 3.3. Comparison between Type IIIa and IIIb Prolapse Modalities

Two types of prolapse, IIIa and IIIb, included the majority of the enrolled patients, with 48 (39.3%) and 52 (42.6%) subjects, respectively. The comparison of these two groups showed that the HDSS was higher in type IIIb, even if the differences were not statistically different (Table 4). Regarding the assessment of the patients’ QoL, it emerged that all four domains of the SHS_HD_ and the total score were significantly worse in group IIIb than in IIIa (Table 4).

## 4. Discussion

Hemorrhoidal disease is traditionally classified using the Goligher classification, which is the most accepted and is almost universally used in clinical practice [3]. Also, the main international guidelines base their diagnostic algorithms on the degree of prolapse assessed according to the Goligher classification [3,4]. However, it has numerous limitations, as summarized by Kuiper et al. [15]: it has never been validated by a study which has certified its ability to stratify HD by its diagnosis and treatment; furthermore, it only considers the prolapse symptom, as assessed by the surgeon; and, finally, it does not evaluate the associated symptoms of HD. In numerous clinical trials, the percentage of patients affected by III- or IV-degree HD can vary from 0 to 100%, indicating either an incorrect classification or a voluntary selection of patients [7]. In addition, the Goligher classification does not take into consideration any other detailed feature of hemorrhoidal prolapse (i.e., frequency and timing—only at defecation or also on other occasions during the day), which, intuitively, could characterize HD’s clinical severity.

As in other diseases, PROMs (patient-reported outcome measures) are becoming more and more widespread in HD management, as they are diagnostic tools which give greater importance to what is reported by patients [12,13]. Their usefulness is not limited only to the diagnostic phase but also to therapeutic planning and could be used in clinical trials to evaluate the outcome of a treatment [15]. Specifically, for HD, hemorrhoidal prolapses seem to be the main symptoms able to condition the therapeutic decision and, when surgery is necessary, the type of procedure to be performed.

For these reasons, our study focused mainly on hemorrhoidal prolapse. The patients enrolled were asked to detail the frequency and timing of presentation of their symptom, and five categories of patients were consequently identified. There was a progressive worsening of all symptoms, not only of the prolapse itself. This approach of investigation of hemorrhoidal prolapses is no longer just graded on the basis of the clinical visit performed by a surgeon in their office, but on the patient’s daily experience. Then, not surprisingly, the patients’ quality of life, as assessed by the SHS score, also significantly worsened in the five patient types identified.

This study showed that 100 out of the 122 enrolled patients could be classified as III-a or III-b according to our new prolapse scoring: in fact, several patients, who would be classified as II-degree according to the Goligher classification, had a disease severity more similar to our new III-a or III-b type.

These data could also have implications for potential therapeutic choices: some of the patients traditionally classified as Goligher I- or II-degree HD may be offered only conservative or outpatient treatments, not in line with the real impact that the disease has on their quality of life and real expectations.

In this study, the discriminating factor in classifying patients as III-a or III-b was the occurrence of hemorrhoidal prolapse only at defecation or even beyond it: all domains of the SHS score as well as the total score were significantly higher when the prolapse occurred even far from defecation, regardless of its manual reducibility. These data are not difficult to understand if we consider how much the patient could be disturbed by the occurrence of a hemorrhoidal prolapse symptom even during normal daily activities (work, physical activity, walking, etc.), with an immediate and evident effect on their quality of life. A previous study by Gerjy et al. [7] instead concluded that there was no correlation between the anatomical grade of HD and the associated symptoms. In this study, however, the authors considered only the manual reducibility of the prolapse, not analyzing the timing of its presentation; moreover, greater importance was given to the evaluation made by the surgeon in their office on the external component of the disease and its reducibility or not, without considering the impact on the patients’ quality of life. On the contrary, we considered that the assessment made by the surgeon can be significantly affected by the patient’s position during the examination and, more importantly, the absence of defecation and normal life activities during the visit. The patient, if accurately asked, can report the occurrence and modality of presentation of their symptoms (in particular concerning the hemorrhoidal prolapse) and give the clinician the immediate opportunity for a correct classification. Of course, the physical examination should confirm the HD features; in particular, concerning hemorrhoidal prolapses, the physical exam must correctly differentiate the skin tags from the real prolapsing hemorrhoids.

The major limitations of our study were the fact that it was a single-center study and the relatively small number of patients enrolled. A further multicenter study is ongoing to overcome these limitations.

## 5. Conclusions

The modality and frequency of hemorrhoidal prolapse have an impact on patients’ quality of life, as assessed with validated tools. By analyzing these characteristics of prolapse, it was possible to identify new types of patients, different from the traditional classifications.

However, it is necessary to evaluate whether the application of this new stratification of patients in clinical practice can improve the choice between the various existing therapeutic approaches and, consequently, patient outcomes.

## Figures and Tables

**Table 1:** Prolapse modality assessment.

Please indicate how your hemorrhoidal prolapse usually occurs:
I never have hemorrhoidal prolapse I have prolapse only at defecation which immediately reduces by itself I have prolapse only at defecation and wait for it to reduce on its own I have prolapse only at defecation which I have to reduce manually I have prolapse both at defecation and also not at defecation but which reduces by itself I have prolapse both at defecation and also not at defecation that I have to manually reduce I have hemorrhoidal prolapse that is always fixed on the outside (I cannot reduce it)

**Table 2:** The Hemorrhoidal Disease Symptom Score (HDSS) and the Short Health Scale HD (SHS_HD_)^11^.

Hemorrhoidal Disease Symptom Score
The following questions deal with symptoms caused by hemorrhoids. Your answers should reflect your symptoms during the last 3 months (1 answer per question):
1. How often do you feel pain from your hemorrhoids? □ Never □ Less than once a month □ Less than once a week □ 1–6 days per week □ Every day (always)
2. How often do you feel itching or discomfort of the anus? □ Never □ Less than once a month □ Less than once a week □ 1–6 days per week □ Every day (always)
3. How often do you bleed when passing stool? □ Never □ Less than once a month □ Less than once a week □ 1–6 days per week □ Every day (always)
4. How often do you soil your underwear (soiling from the anus)? □ Never □ Less than once a month □ Less than once a week □ 1–6 days per week □ Every day (always)
5. How often do you feel a swelling or a prolapsing hemorrhoid? □ Never □ Less than once a month □ Less than once a week □ 1–6 days per week □ Every day (always)
**Short Health Scale_HD_**
The following questions deal with how your symptoms caused by hemorrhoids affect your daily life (one answer per question):
1. In your view, how severe are your symptoms caused by hemorrhoids? Please grade your symptoms on a 7-point scale, where 1 is “no symptoms” and 7 is “severe symptoms”.
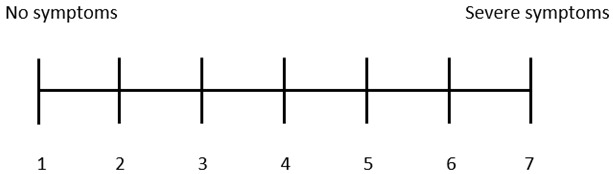
2. Do your symptoms interfere with your daily activities? Please grade your answer on a 7-point scale, where 1 is “not at all” and 7 is “interfere to a very high degree”.
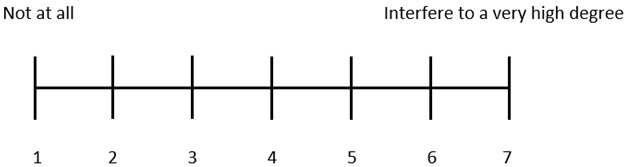
3. Do your symptoms cause much concern? Please grade your answer on a 7-point scale, where 1 is “no concerns” and 7 is “constant concerns”.
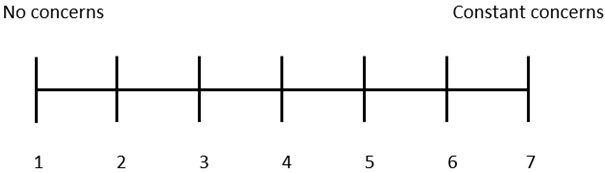
4. How is your general feeling of well-being? Please grade your answer on a 7-point scale, where 1 is “very good” and 7 is “very bad”.
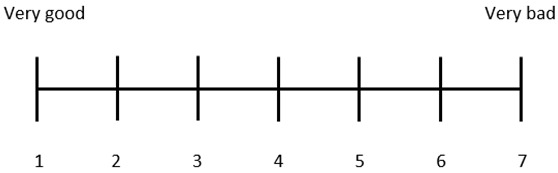

**Table 3:** Results.

Age (mean, SD)	
<50 years	59 (48.4)
>50 years	63 (51.6)
**Gender (n, %)**	
female	53 (43.4)
male	69 (56.6)
**Bristol stool scale (n, %)**	
1	11 (4.1)
2	13 (10.7)
3	35 (28.7)
4	31 (25.4)
5	17 (13.9)
6	15 (12.3)
7	0
**HDSS score (mean, SD)**	
pain	2.1 (1.5)
itching	1.9 (1.4)
bleeding	2.2 (1.2)
soiling	0.6 (1.1)
prolapse/swelling	3.1 (1.2)
**TOTAL score**	9.8 (3.3)
**SHS QoL (mean, SD)**	
Severity symptoms	5.0 (1.5)
Interference	4.5 (1.9)
Concern	4.9 (1.6)
General feeling	4.2 (1.7)
**TOTAL score**	18.6 (5.2)
**PROLAPSE MODALITY (n, %)**	
answer # 1	5 (4.1)
answer # 2	9 (7.4)
answer # 3	23 (18.9)
answer # 4	25 (20.5)
answer # 5	22 (18.0)
answer # 6	30 (24.6)
answer # 7	8 (6.6)
**PATIENTS’ CLASSIFICATION (n, %)**	
type I	5 (4.1)
type II	9 (7.4)
type IIIa	48 (39.3)
type IIIb	52 (42.6)
type IV	8 (6.6)

HDSS score = the Hemorrhoidal Disease Symptom Score; SHS = the Short Health ScaleHD.

**Table 4:** Comparison between type IIIa and IIIb prolapse modalities.

	TYPE IIIa	TYPE IIIb	*p*-Value
**Age**	52.4 (13.7)	46.4 (10.6)	**0.012**
**Gender (n, %)**			**0.021**
female	14 (29.2)	27 (51.9)	
male	34 (70.8)	24 (48.1)	
**Bristol stool scale (n, %)**			0.809
1	3 (6.3)	5 (9.6)	
2	4 8.3)	7 (13.5)	
3	15 (31.3)	15 (28.8)	
4	15 (31.3)	12 (23.1)	
5	6 12.5)	5 (9.6)	
6	5 (10.4)	8 (15.4)	
7	0	0	
**HDSS score (mean, SD)**			
pain	1.8 (1.5)	2.2 (1.5)	0.311
itching	1.8 (1.5)	1.9 (1.3)	0.794
bleeding	2.4 (1.1)	2.1 (1.2)	0.271
soiling	0.7 (1.1)	0.6 (1.1)	0.916
prolapse/swelling	3.2 (1.0)	3.5 (0.6)	0.106
**TOTAL HDSS score**	9.9 (2.7)	10.3 (3.2)	0.747
**SHS QoL (mean, SD)**			
Severity symptoms	4.5 (1.5)	5.4 (1.2)	**0.004**
Interference	4.2 (1.9)	5.0 (1.4)	**0.022**
Concern	4.5 (1.5)	5.4 (1.5)	**0.003**
General feeling	3.7 (1.6)	4.7 (1.6)	**0.004**
**TOTAL score**	16.8 (5.1)	20.5 (4.0)	**0.001**

HDSS score = the Hemorrhoidal Disease Symptom Score; SHS = the Short Health ScaleHD.

## Data Availability

The original contributions presented in the study are included in the article, further inquiries can be directed to the corresponding author.
